# Extent of Cytomegalovirus Replication in the Human Host Depends on Variations of the HLA-E/UL40 Axis

**DOI:** 10.1128/mBio.02996-20

**Published:** 2021-03-16

**Authors:** Hannes Vietzen, Timo Rückert, Svenja Hartenberger, Claudia Honsig, Peter Jaksch, Silvana Geleff, Quirin Hammer, Chiara Romagnani, Maia Segura-Wang, Elisabeth Puchhammer-Stöckl

**Affiliations:** a Center for Virology, Medical University of Vienna, Vienna, Austria; b Innate Immunity, German Rheumatism Research Center, Leibniz Association, Berlin, Germany; c Division of Clinical Virology, Medical University of Vienna, Vienna, Austria; d Division of Thoracic Surgery, Medical University of Vienna, Vienna, Austria; e Clinical Institute of Pathology, Medical University of Vienna, Vienna, Austria; f Center for Infectious Medicine, Department of Medicine Huddinge, Karolinska Institutet, Karolinska University Hospital, Stockholm, Sweden; Medical School, National and Kapodistrian University of Athens

**Keywords:** human cytomegalovirus, NK cells, HLA-E, UL40, lung transplantation, NKG2A, NKG2C

## Abstract

Human cytomegalovirus (HCMV) may cause severe infections in lung transplant recipients (LTRs). In response to HCMV infections, a subset of NKG2C^+^ NK cells expands, which limits HCMV replication and is characterized by high expression of the activating NKG2C/CD94 and absence of the inhibitory NKG2A/CD94 receptor. Both receptors bind to HLA-E, which is stabilized by HCMV-encoded UL40 peptides. HLA-E and UL40 occur as different genetic variants. In this study, we investigated the interplay between the human NK cell response and the infecting HCMV-UL40 strain, and we assessed the impact of HCMV-UL40 and of donor- and recipient-encoded HLA-E*0101/0103 variants on HCMV replication after lung transplantation. We included 137 LTRs displaying either no or low- or high-level (>1,000 copies/ml plasma) viremia. HCMV-UL40 and HLA-E*0101/0103 variants were determined. UL40 diversity was investigated by next-generation sequencing. UL40 peptide-dependent NK cell cytotoxicity was assessed by flow cytometry. Donor-encoded HLA-E*0101/0103 was significantly associated with development of high-level viremia after transplantation (*P* = 0.007). The HCMV-UL40 variant VMAPRTLIL occurred significantly more frequently in highly viremic LTRs, and the variant VMTPRTLIL occurred significantly more frequently in low-viremic LTRs (*P* = 0.004). This difference was associated with a better inhibition of NKG2A^+^ NKG2C^−^ NK cells by VMAPRTLIL (*P* < 0.001). In LTRs with repeated high-level viremic episodes, HCMV strains with UL40 variants displaying low affinity to the patients’ HLA-E variant emerged over time. The HLA-E-UL40 axis has a substantial impact on the level of HCMV replication in LTRs. The interplay between UL40 peptide variants, the recipient HLA-E status, and the activation of inhibitory NKG2A^+^ NKG2C^−^ cells is of major importance for development of high-level viremia after lung transplantation.

## Abstract (precis)

Infection with human cytomegalovirus (HCMV) is associated with substantial morbidity in immunosuppressed patients and after congenital infections. Therefore, development of a vaccine against HCMV is a main public health priority.

## INTRODUCTION

Human cytomegalovirus (HCMV) may cause severe infections in lung transplant recipients (LTRs) (1, 2). The course of HCMV replication and disease is dependent on the donor (D) and recipient (R) serostatus, and D^+^/R^−^ LTRs especially may undergo high-level virus replication during primary HCMV infection. In seropositive recipients (R^+^), HCMV reactivation and reinfections may occur.

HCMV-specific immune responses limit the viral spread in LTRs but cannot eliminate the virus (3). This results in a dynamic interplay between host immune responses and HCMV immune-evasive mechanisms which may shape the HCMV populations emerging in the individual host (4, 5). Natural killer (NK) cells play a key role in HCMV-specific immune defense. NK cell activation depends on activating or inhibitory receptors, and in response to HCMV, a subset of NKG2C^+^ NK cells expands, showing elevated expression of the activating CD94/NKG2C and absent expression of the inhibitory CD94/NKG2A receptor (6, 7). Activation, direct cytotoxicity, and release of effector molecules of NKG2C^+^ cells against HCMV-infected cells is mediated by interaction of NKG2C and its cellular ligand HLA-E (8–10).

The stable expression of HLA-E on the surface of HCMV-infected cells depends on a peptide encoded by the polymorphic HCMV-UL40 region (11, 12). Distinct variants of this UL40 peptide occur, which could have impact on the peptide binding affinities to HLA-E molecules and may subsequently alter NK cell activation and effector functions (11, 13). UL40 peptide presentation may be influenced by the presence of naturally occurring genetic variants of HLA-E. HLA-E occurs in European populations mostly as HLA‐E*0101 or HLA‐E*0103 (14), which show significantly different cell surface expression levels and peptide binding capacities (14, 15).

The aim of the present study was to assess the role of the HLA-E-UL40 axis in the HCMV defense after lung transplantation and to identify whether human and viral genetic variations thereof have a substantial impact on the extent of posttransplant HCMV replication. The present data show for the first time that the interaction between the individual HCMV UL40 peptide variant, HLA presentation, and NK cell responses has significant impact on the extent of virus replication also in the clinical situation after transplantation.

## RESULTS

### Characteristics of the study cohort.

The study cohort consisted of 137 patients, of whom 68 LTRs (49.6%) developed at least one episode of high-level HCMV replication (>1,000 copies/ml plasma), while in 69 LTRs (50.4%) either no (*n* = 38) or only low-level HCMV replication (*n* = 31, <1,000 copies/ml plasma) was detected within the 1- (R^+^) or 2-year (D^+^/R^−^) follow-up. The patient groups are presented in Table 1.

**TABLE 1:** Characteristics of the study cohort^*b*^

	No/low viral load (<1,000 copies/ml) (*n* = 69)	Viremia (>1,000 copies/ml) (*n* = 68)	*P* value^*a*^
Gender (% female)	*n* = 29 (42%)	*n* = 36 (53%)	ns

Median age, yr (range)	57.9 (19–66)	53.5 (18–71)	ns

D/R serostatus			ns
D^+^/R^−^	*n* = 17 (24.6%)	*n* = 19 (28.0%)	
D^−^/R^+^	*n* = 24 (34.8%)	*n* = 13 (19.1%)	
D^+^/R^+^	*n* = 28 (40.6%)	*n* = 36 (52.9%)	

Viral load (copies/ml plasma) (min–max)			
*n* (%)	*n* = 31 (44.9%)		
Median days post-LTX (min–max)	150 (65–319)		
Median viral load (copies/ml plasma) (min–max)	141 (126–454)		

1st highly viremic episode			
*n* (%)		*n* = 68 (100%)	
Median days post-LTX (min–max)		141 (26–454)	
Median viral load (copies/ml plasma) (min–max)		4.3 × 10^3^ (1,400–1.4 × 10^6^)	

2nd highly viremic episode			
*n* (%)		*n* = 25 (36.8%)	
Median days post-LTX (min–max)		194 (113–587)	
Median viral load (copies/ml plasma) (min–max)		2.7 × 10^3^ (1,060–9.2 × 10^4^)	

3rd highly viremic episode			
*n* (%)		*n* = 2 (2.9%)	
Mean days post-LTX (min–max)		370 (113–587)	
Mean viral load (copies/ml plasma) (min–max)		1.5 × 10^3^ (1,240–3,130 × 10^3^)	

4th highly viremic episode			
*n* (%)		*n* = 1 (1.5%)	
Days post-LTX		492	
Viral load (copies/ml plasma)		1.1 × 10^3^	

a Differences between groups were assessed with the Mann-Whitney or χ^2^ test.

b Abbreviations: D^+^, HCMV-seropositive donor; D^−^, HCMV-seronegative donor; ns, not significant; R^+^, HCMV-seropositive recipient; R^−^, HCMV-seronegative recipient; LTX, lung transplantation.

### HLA-E variants and HCMV replication.

**FIG 1:**
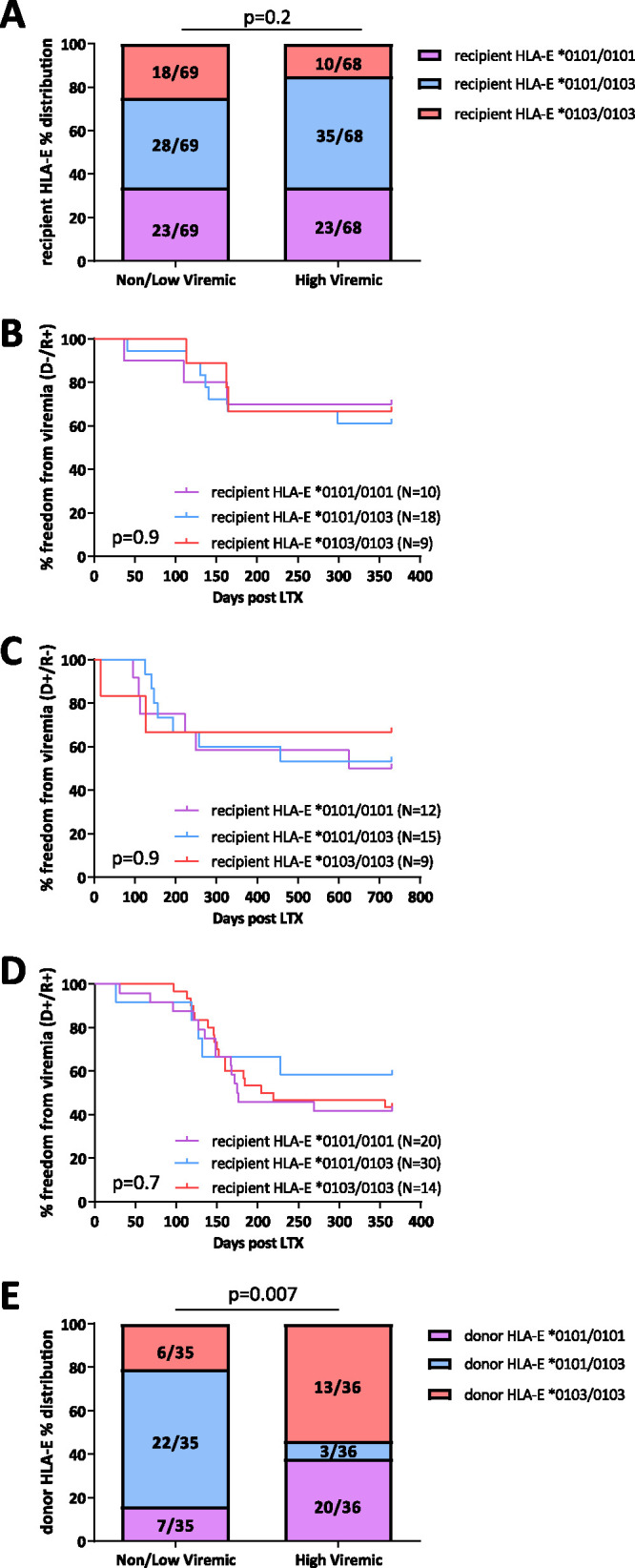
(A) Distributions of LTR HLA-E variants between non-/low-viremic and highly viremic lung transplant recipients (LTRs). Bars represent the relative frequency of HLA-E*0101/0101, HLA-E*0101/0103, and HLA-E*0103/0103 for each group. χ^2^ test was used for statistical comparison between variants. (B to D) Kaplan-Meier curves for the freedom from viremia. Curves represent HLA-E*0101/0101, HLA-E*0101/0103, and HLA-E*0103/0103 variants in D^−^/R^+^ (B), D^+^/R^−^ (C), and D^+^/R^+^ (D) groups. Survival curves were compared with the Mantel-Cox test. (E) Distributions of lung transplant donor (LTD) HLA-E variants between non-/low-viremic and highly viremic LTRs. Bars represent the relative frequency of HLA-E*0101/0101, HLA-E*0101/0103, and HLA-E*0103/0103 for each group. χ^2^ test was used for statistical comparison between variants. D/R, donor and recipient serostatus.

We then investigated whether the recipient or donor HLA-E variant was associated with development of high-level viremia. No significant association was found between recipient HLA-E variant and high-level viremia (Fig. 1A to D). Analysis of donor HLA-E variants from the formalin-fixed paraffin-embedded (FFPE) lung samples revealed that the heterozygous HLA-E*0101/0103 variant in the donor lung was significantly overrepresented in LTRs with no/low-level HCMV replication. High-level viremia developed more frequently when donor lungs carried homozygous HLA-E*0101 and HLA-E*0103 variants (Fig. 1E). This association was observed in D^+^ patients only (see Fig. S1 in the supplemental material).

10.1128/mBio.02996-20.1 FIG S1 Distributions of donor HLA-E variants between non-/low-viremic and highly viremic lung transplant recipients (LTRs). Bars represent the relative frequency of HLA-E*0101/0101, HLA-E*0101/0103, and HLA-E*0103/0103 variants in D^−^/R^+^ (A), D^+^/R^−^ (B), and D^+^/R^+^ (C) groups. χ^2^ test was used for statistical comparison between variants. D/R, donor and recipient serostatus. Download FIG S1, PDF file, 0.05 MB. Copyright © 2021 Vietzen et al. 2021 Vietzen et al. https://creativecommons.org/licenses/by/4.0/ This content is distributed under the terms of the Creative Commons Attribution 4.0 International license.

### Analysis of the patients’ HCMV UL40 peptide variants.

**FIG 2:**
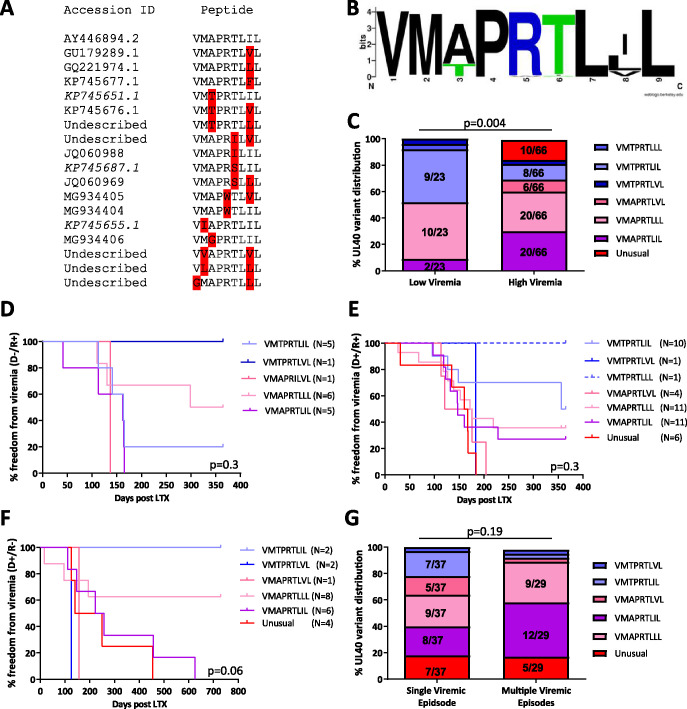
(A) Sequence alignment of the 18 detected UL40 strains. Variations from the consensus sequence in the DNA and peptide sequence are highlighted in red. (B) Sequence logo alignment of the relative frequency of 90 sequenced UL40 strains. Sequence logos were created with the web tool of the University of California (https://weblogo.berkeley.edu/). (C) Distributions of the six most frequent UL40 variants and the combined “unusual” variant between non-/low-viremic and highly viremic lung transplant recipients (LTR). Bars represent the relative frequency of UL40 strains in each group. χ^2^ test was used for statistical comparison between variants. (D to F) Kaplan-Meier curves for the freedom from viremia. Curves represent the six most frequent UL40 variants and the combined “unusual” variant in D^−^/R^+^ (D), D^+^/R^+^ (E), and D^+^/R^−^ (F) groups. Survival curves were compared with the Mantel-Cox test. (G) Distributions of the five most frequent UL40 variants and the combined “unusual” variant between patients with a single viremic episode or multiple viremic episodes (>1,000 copies/ml blood) in a 1- (R^+^) or 2-year (D^+^/R^−^) follow-up. Bars represent the relative frequency of UL40 strains in each group. χ^2^ test was used for statistical comparison between variants. D/R, donor and recipient serostatus; LTR, lung transplant recipient.

Next, we analyzed the UL40 peptide sequences of the HCMV strains detected in the patients. Sequences of the HCMV UL40 gene could be identified from the plasma of 90 LTRs, including 24 patients with low-level viremia. UL40 sequences of the patients showed two polymorphic hot spots at the 3rd and 8th amino acid residues (Fig. 2A and B), resulting in the variants VMAPRTLIL (*n* = 22; 24.4%), VMAPRTLLL (*n* = 29; 32.2%), VMAPRTLVL (*n* = 6; 6.6%), VMTPRTLIL (*n* = 17; 18.8%), VMTPRTLVL (*n* = 3; 3.3%), and VMTPRTLLL (*n* = 1).

Comparison of the individual peptide variants between the patient groups showed that the VMTPRTLIL variant was more frequently present in patients with low viremia while the VMAPRTLIL variant was overrepresented in high-level viremic patients (*P* = 0.004, F-test, Fig. 2C).

In addition, 12 (13.3%) patients showed “unusual” variants, with variations in conserved amino acid residues (Fig. 2A and B). As shown in Fig. 2C to F, these variants were exclusively found in highly viremic D^+^ patients, albeit some peptides were found in a low number of patients.

We further compared the viral loads between highly viremic patients infected with HCMV strains containing the VMAPRTLIL (median: 3,740 copies/ml; range: 1,400 to 1.26 × 10^6^ copies/ml) and VMTPRTLIL (median: 1,870 copies/ml; range: 1,400 to 5,480 copies/ml) variants and the unusual variants (median: 5,360 copies/ml; range: 1,620 to 3.54 × 10^6^ copies/ml). LTRs with the VMTPRTLIL variant showed a lower viral load than patients with the VMAPRTLIL or unusual variants (both: *P* = 0.04, Dunn’s test).

We then assessed whether distinct UL40 peptide variants were associated with development of repeated episodes of high-level viremia. Therefore, the occurrence of the UL40 variants in patients with a single highly viremic episode (*n* = 37; 56%) was compared to that in patients with more than one episode (*n* = 29; 44%) in the follow-up (Fig. 2G). Patients carrying the VMTPRTLIL variant were less likely to develop more than one viremic episode in the follow-up than those with the VMAPRTLIL variant (*P* = 0.03, F-test).

### Distinct UL40 variants are associated with NK cell activity.

**FIG 3:**
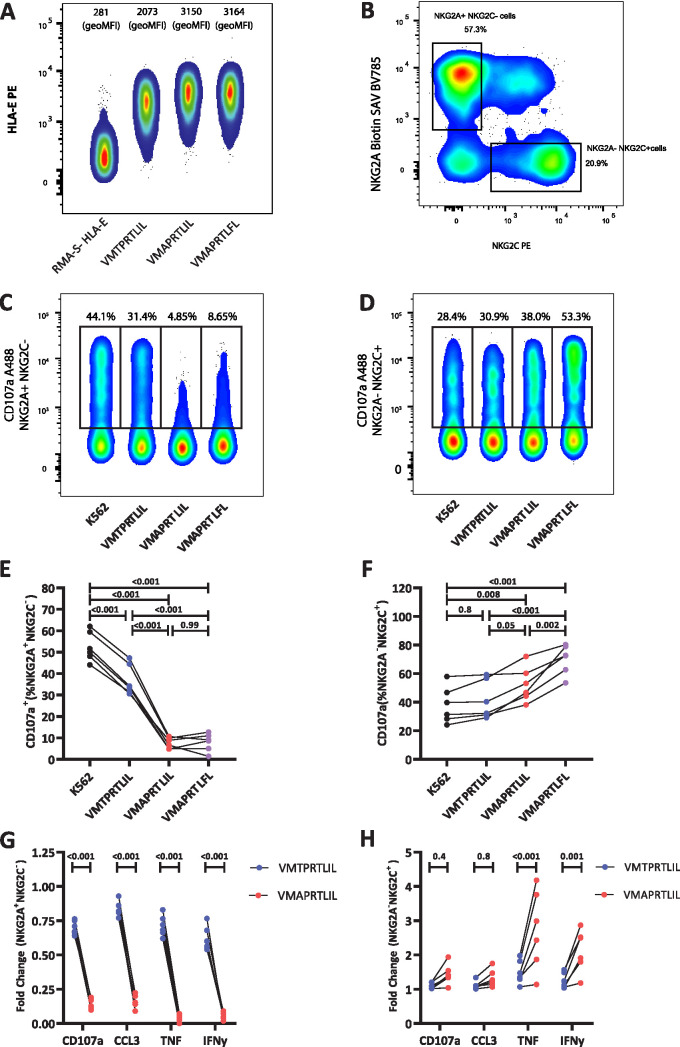
(A) Representative examples and geometric mean fluorescence intensities (geoMFIs) of VMTPRTLIL-, VMAPRTLIL-, or VMAPRTLFL-induced HLA-E surface stabilization on peptide-pulsed RMA-S–HLA-E cells. (B) Representative gating strategy for NKG2A^−^ NKG2C^+^ and NKG2A^+^ NKG2C^−^ NK cells. (C to F) Representative staining (C and D) and quantification of degranulation (E and F) of NKG2A^+^ NKG2C^−^ (C and E) and NKG2A^−^ NKG2C^+^ (D and F) NK cells in response to K562/HLA-E cells pulsed with the indicated peptides. (G and H) Fold change of the indicated effector functions normalized to the response toward unpulsed K562/HLA-E cells. Two-way ANOVA with Sidak *post hoc* test (E and F) or paired *t* test (G and H) was used for statistical comparison between the UL40 variants.

As the VMAPRTLIL variant was significantly overrepresented in highly viremic patients compared to VMTPRTLIL, we further analyzed the capacity of these two UL40 peptide variants to stabilize HLA-E on the surface of target cells and to modulate activation of NKG2C^+^ NKG2A^−^ and NKG2C^−^ NKG2A^+^ NK cells. Incubation with VMTPRTLIL led to a clear but somewhat lower stabilization of HLA-E (Fig. 3A). We then tested the response of NKG2C^+^ NKG2A^−^ and NKG2C^−^ NKG2A^+^ cells toward K562/HLA-E target cells pulsed with the peptides VMTPRTLIL, VMAPRTLIL, and VMAPRTLFL, the last serving as a control for activation through NKG2C, as previously described (11). Unpulsed K562/HLA-E cells induced an intermediate response of both NK cell subsets, as measured by induction of degranulation (CD107) and cytokine production (gamma interferon [IFN-γ], tumor necrosis factor [TNF], CCL3), enabling us to assess both inhibitory signals induced by engagement of NKG2A and activating effects via NKG2C (Fig. 3C to H and Fig. S2 and S3). VMAPRTLIL was very efficient in inhibiting NKG2A^+^ NKG2C^−^ NK cells, leading to an almost complete blockade of all effector functions of this subset. Interestingly, pulsing with VMTPRTLIL resulted in only a modest decrease of NKG2A^+^ NKG2C^−^ NK cell activation. VMAPRTLIL also induced a slightly enhanced response of NKG2A^−^ NKG2C^+^ NK cells, especially for TNF-α and IFN-γ, but this activating effect was much less pronounced than for the strongly activating VMAPRTLFL (Fig. 3C, E, and G; Fig. S3). NKG2A^−^ NKG2C^+^ NK cells responded almost equally to unpulsed and VMTPRTLIL-pulsed target cells (Fig. 3D, F, and H; Fig. S3). Overall, this indicates that VMTPRTLIL is not very efficient in engaging NKG2A, thereby mainly lacking the inhibitory functions exerted by VMAPRTLIL.

10.1128/mBio.02996-20.2 FIG S2 Representative example of the gating strategy and determination of viable, CD56^+^ NKG2A^+^ NKG2C^−^ or CD56^+^ NKG2A^−^ NKG2C^+^ NK cells. Download FIG S2, PDF file, 0.5 MB. Copyright © 2021 Vietzen et al. 2021 Vietzen et al. https://creativecommons.org/licenses/by/4.0/ This content is distributed under the terms of the Creative Commons Attribution 4.0 International license.

10.1128/mBio.02996-20.3 FIG S3 Representative staining (A and C) and quantification of effector functions (B and D) of NKG2A^+^ NKG2C^−^ (A and B) and NKG2A^−^ NKG2C^+^ (C and D) NK cells in response to K562/HLA-E cells pulsed with the indicated peptides. Download FIG S3, PDF file, 1.4 MB. Copyright © 2021 Vietzen et al. 2021 Vietzen et al. https://creativecommons.org/licenses/by/4.0/ This content is distributed under the terms of the Creative Commons Attribution 4.0 International license.

### Analysis of the kinetics of UL40 variant populations by NGS.

We further questioned whether HCMV strains with different UL40 peptide sequences may circulate simultaneously in a patient, and whether strains including specific UL40 variants exhibit different kinetics over time in association with the patients’ HLA-E alleles. For this purpose, we selected all LTRs (*n* = 29) with more than one highly viremic episode and analyzed plasma samples from each episode for HCMV-UL40 by next-generation sequencing (NGS). The results are shown in Tables 2 to 4 together with the patients’ HLA-E variants. In 17 LTRs (58.6%), a single UL40 variant was found in all episodes (Table 2). Three patients (12%), all D^+^/R^+^, exhibited a single UL40 variant in the plasma in the first viremic episode, and this was replaced by another variant in the second episode (Table 3). To further assess the relationship between the UL40 variant and the patients’ HLA-E, we predicted binding affinities of the UL40 peptide variants to the patients’ HLA-E*0101/0101 and HLA-E 0103/0103 using the Immune Epitope Database (IEDB) analysis resource stabilized matrix method (SMM) tool (Table S2). The UL40 variants found during the second viremic episode were not altered toward lower peptide binding affinity to the donor or recipient HLA-E variant. Finally, in 9 patients (36%), a mixture of different UL40 variant strains was simultaneously detectable in the plasma at the first highly viremic episode, but remarkably, in all cases only a single UL40 variant was still detectable in the second episode (Table 4). In all four LTRs who displayed a homozygous HLA-E variant (patient [Pat] identifiers [ID] 21 to 23 and 29), the UL40 peptide with the respective lowest peptide binding affinity to the patient’s HLA-E variant emerged in the second viremic episode, as shown in Table 4. All of these were “unusual” variants (Table S2). In contrast, in LTRs heterozygous for HLA-E*0101/0103, it was always the most abundant UL40 peptide variant observed in the first viremic episode, which emerged in the second highly viremic episode (Pat ID 24 to 28).

**TABLE 2:** Single UL40 strains^*a*^

Patient ID	D/R serostatus	HLA-E genotype	UL40 1st viremic episode	UL40 2nd viremic episode
1	D^+^/R^−^	R: *0101/0101	VMTPRTLVL	VMTPRTLVL
2	D^+^/R^+^	R: *0101/0103	VMAPRTLIL	VMAPRTLIL
3	D^+^/R^−^	R: *0101/0103	VIAPRTLIL	VIAPRTLIL
4	D^+^/R^+^	R: *0101/0103	VMAPRILLL	VMAPRILLL
5	D^+^/R^+^	R: *0101/0101	VMAPRTLLL	VMAPRTLLL
6	D^+^/R^−^	R: *0101/0103	VMAPRTLVL	VMAPRTLVL
7	D^+^/R^−^	R: *0101/0103	VMAPRTLIL	VMAPRTLIL
8	D^+^/R^+^	R: *0101/0103	VMAPRTLIL	VMAPRTLIL
9	D^+^/R^+^	R: *0101/0103	VMAPRTLIL	VMAPRTLIL
10	D^+^/R^+^	R: *0101/0103	VMAPRTLIL	VMAPRTLIL
11	D^+^/R^−^	R: *0101/0103	VMAPRTLIL	VMAPRTLIL
12	D^−^/R^+^	R: *0101/0103	VMAPRTLLL	VMAPRTLLL
13	D^+^/R^−^	R: *0103/0103	VMAPRTLIL	VMAPRTLIL
14	D^+^/R^−^	R: *0103/0103	VMAPRTLIL	VMAPRTLIL
15	D^+^/R^−^	R: *0103/0103	VMAPRTLLL	VMAPRTLLL
16	D^+^/R^+^	R: *0103/0103	VMAPRTLLL	VMAPRTLLL
17	D^+^/R^+^	R: *0103/0103	VMAPRTLLL	VMAPRTLLL

a Abbreviations: D^+^, HCMV-seropositive donor; D^−^, HCMV-seronegative donor; R^+^, HCMV-seropositive recipient; R^−^, HCMV-seronegative recipient; V, viremic episode.

**TABLE 3:** UL40 sequence adaptions^*a*^

Patient ID	D/R serostatus	HLA-E genotype	UL40 V1 (SMM IC_50_ [nM])	UL40 V2 (SMM IC_50_ [nM])
18	D^+^/R^+^	R: *0101/0101 D: *0101/0101	VLAPRTLLL (0101: 111.5)	VMTPRTLIL (0101: 60.8)
19	D^+^/R^+^	R: *0103/0103 D: *0103/0103	VMTPRTLIL (0103: 8,674.4)	VMAPRTLIL (0103: 10,333.3)
20	D^+^/R^+^	R: *0103/0103 D: *0101/0101	VMAPRTLLL (0101: 45; 0103: 10,333.3)	VMAPRTLVL (0101: 45; 0103: 10,333.3)

a Abbreviations: D^+^, HCMV-seropositive donor; D^−^, HCMV-seronegative donor; R^+^, HCMV-seropositive recipient; R^−^, HCMV-seronegative recipient; V, viremic episode. IC_50_, 50% inhibitory concentration.

**TABLE 4:** Mixed UL40 infections^*a*^

Patient ID	D/R serostatus	HLA-E genotype	UL40 V1 (SMM IC_50_ [nM])	UL40 V2 (SMM IC_50_ [nM])
21	D^+^/R^−^	R: *0101/0101 D: *0101/0101	**36.5%** VMGPRTLIL (**0101: 98.9; 0103: 11,021.5)**	VMGPRTLIL (**0101: 98.9; 0103: 11,021.5)**
			23.1% VMAPRTLLL (0101: 45.1; 0103: 10,333.3)	
			21.5% VMAPRTLIL (0101: 47.7; 0103: 10,333.3)	
			15.4% VMTPRTLIL (0101: 60.8; 0103: 8,674.41)	

22	D^+^/R^+^	R: *0101/0101 D: *0101/0103	56.6% VMAPRTLLL (0101: 45.1; 0103: 10,333.3)	VMALRTLLL **(0101: 170.3; 0103: 13,008.9)**
			19.5% VMTPRTLIL (0101: 60.8; 0103: 8,674.4)	
			13.9% VMTPRTLLL (0101: 57.4; 0103: 8,674.4)	
			8.0% VMAPRTLIL (0101: 47.7; 0103: 10,333.3)	
			**1.4%** VMALRTLLL **(0101: 170.3; 0103: 13,008.9)**	

23	D^+^/R^+^	R: *0101/0101 D: *0103/0103	**76.6%** VMAPWTLVL **(0101: 62.1; 0103: 9,252.1)**	VMAPWTLVL **(0101: 62.1; 0103: 9,252.1)**
			17.1% VMAPRTLVL (0101: 45; 0103: 10,333.3)	
			1.6% VMAPRTLIL (0101: 47.8; 0103: 10,333.3)	

24	D^+^/R^+^	R: *0101/0103 D: *0101/0101	**95.8%** VMAPRTLIL **(0101: 47.78; 0103: 10,333.32)**	VMAPRTLIL **(0101: 47.78; 0103: 10,333.32)**
			1.4% VVAPRTLIL (0101: 166.8; 0103: 14,003.6)	

25	D^+^/R^+^	R: *0101/0103 D: *0103/0103	**95.7%** VMAPRTLIL **(0101: 47.78; 0103: 10,333.3)**	VMAPRTLIL **(0101: 47.78; 0103: 10,333.32)**
			1.4% VVAPRTLIL (0101: 166.8; 0103: 14,003.6)	

26	D^−^/R^+^	R: *0101/0103 D: *0101/0101	**76.7%** VMAPRTLIL **(0101: 47.78; 0103: 10,333.3)**	VMAPRTLIL **(0101: 47.78; 0103: 10,333.32)**
			20.7% VMTPRTLIL (0101: 60.8; 0103: 8,674.4)	

27	D^−^/R^+^	R: *0101/0103 D: *0101/0101	**91.8%** VMAPRTLIL **(0101: 47.78; 0103: 10,333.3**)	VMAPRTLIL **(0101: 47.78; 0103: 10,333.32)**
			4.3% VMAPRTLIP (0101: 85.94; 0103: 25,600)	

28	D^−^/R^+^	R: *0101/0103 D: *0101/0101	**94.7%** VMAPRTLLL **(0101: 45; 0103: 10,333.3**)	VMAPRTLLL **(0101: 45; 0103: 10,333.3)**
			1.1% VMAPRTLVP (0101: 80.95; 0103: 25,600)	

29	D^−^/R^+^	R: *0103/0103 D: *0103/0103	56.0% VMAPRTLLL (0101: 45.1; 0103: 10,333.32)	VMGPRTLLL **(0101: 93.37; 0103: 11,021.5)**
			**41.4%** VMGPRTLLL **(0101: 93.37; 0103: 11,021.5)**	

a Abbreviations: D^+^, HCMV-seropositive donor; D^−^, HCMV-seronegative donor; R^+^, HCMV-seropositive recipient; R^−^, HCMV-seronegative recipient; V, viremic episode. Bold indicates the UL40 encoding HCMV strain during the second highly viremic episode.

10.1128/mBio.02996-20.5 TABLE S2 Predicted UL40-HLA-E affinities. Download Table S2, DOCX file, 0.01 MB. Copyright © 2021 Vietzen et al. 2021 Vietzen et al. https://creativecommons.org/licenses/by/4.0/ This content is distributed under the terms of the Creative Commons Attribution 4.0 International license.

## DISCUSSION

In the present study, we demonstrate that the HLA-E-UL40 axis is strongly associated with immune defense against HCMV and that naturally occurring variations of its components have a substantial impact on the extent of HCMV replication in the human host.

A major finding of this study was that specific variations of the viral UL40 peptide are associated with a significantly higher level of HCMV replication. The UL40 peptide variant VMAPRTLIL was significantly more prevalent in patients developing high-level virus replication, whereas in patients who developed only low-level and self-resolving HCMV viremia, the VMTPRTLIL variant was especially identified. While in previous *in vitro* studies among others the VMAPRTLIL peptide was subject to investigation (11, 13), the VMTPRTLIL peptide has never been analyzed so far.

When we assessed the functional difference between the two peptides, it became apparent that VMTPRTLIL induced much less inhibition via NKG2A than VMAPRTLIL, which engaged very efficiently inhibitory NKG2A^+^ NK cells and thereby limited the antiviral response against the HCMV strains displaying this peptide variant. Differences between VMTPRTLIL and VMAPRTLIL in the activation via NKG2C were less significant. Thus, the higher level of virus replication in patients with HCMV strains carrying the UL40 peptide VMAPRTLIL can be traced back in particular to an increase of NK cell inhibitory signals upon presentation of this variant. Immune pressure against peptide-induced activating signals seems to be less relevant in this range of signal strength but seems to be part of the equation for high-affinity ligands for NKG2C, as judged by the low frequency of the VMAPRTLFL variant observed in this and other studies (11).

From a structural point of view, nonameric peptides are mainly anchored in the HLA-E peptide groove by interactions of the amino acid side chains at position 2, 7, and 9, which are deeply buried into pockets formed by HLA-E (16). In addition, the side chain of position 3 interacts with HLA-E, but the corresponding binding pocket is rather shallow. Our observation of a somewhat lower stabilization of HLA-E by VMTPRTLIL suggests that threonine fits only imperfectly into this pocket, which is also supported by a systematic screening of a partially randomized peptide library for HLA-E binding, in which threonine was not a preferred amino acid at position 3 (17). As position 3 is not solvent exposed, this suggests that either the suboptimal stabilization or an altered conformation of other peptide residues is the underlying reason for the functional differences we observed.

Interestingly, also the UL40 variants exhibiting rare and unusual structures were significantly associated with the development of high-level viremia. These findings await further detailed analyses.

The UL40 peptide is presented by HLA-E, and distinct UL40 peptides show different binding affinities to the HLA-E variants occurring in humans, which may further affect lysis by NKG2C^+^ NK cells (13). Our data show that the presence of the heterozygous HLA-E*0101/0103 variant in the HCMV-positive allografts was significantly associated with the occurrence of no/low-level virus replication after transplantation. HLA-E*0101 and HLA-E*0103 variants differ only in amino acid residue 109, which affects HLA-E surface levels and peptide binding capacities (14, 15). It was speculated that the maintenance of two HLA-E alleles in heterozygous individuals may be a selective advantage by providing a broader spectrum of high-affinity peptides (18–20). The present data suggest that the heterozygous HLA-E graft may better control the initial replication of HCMV in the lung and thus limit subsequent viral spread.

Patients may host simultaneously more than one HCMV-strain (21), and it is still an open question which is the selection mechanism determining which HCMV strains evolve in the human host over time. Our data suggest that the interplay between host HLA-E variants and distinct viral UL40 variant populations may play a role in determining which HCMV strains emerge over time in an infected person. In all homozygous HLA-E*0101 or HLA-E*0103 transplant recipients, the UL40 variant with the lowest calculated affinity toward the patient’s HLA-E alleles evolved in the follow-up. Thus, it appears that especially infected cells carrying UL40 strains with high affinity to the respective HLA-E alleles may be cleared by the human NKG2C^+^ NK cell response. Thus, our data further support the hypothesis that polymorphisms in the UL40 peptide provide a potential HCMV-specific immunoevasive mechanism (22). However, further extended analyses will be needed to confirm the present findings.

The selection of reemerging HCMV strains over time in heterozygous HLA-E*0101/0103 hosts is yet unclear. In our heterozygous HLA-E*0101/0103 recipients, especially peptide variants which show a low predicted binding affinity to HLA-E*0103/0103 predominated. It was previously demonstrated that the peptide-bound HLA-E*0103/0103 variant is overall more stable on the cell surface than HLA-E*0101/0101 (15). Thus, it is feasible that also in HLA-E*0101/0103 individuals, selection toward HLA-E*0103/0103 low-affinity peptides may possibly occur.

In summary, the present study revealed that distinct variants of the HCMV UL40 peptide are specifically associated with development of high-level HCMV replication in the human host and that this is due to differences in the activation of inhibitory NKG2A^+^ cells in response to the peptide. The data also suggest that UL40 peptide variations may develop toward low affinity to the recipient homozygous HLA-E status, but this finding awaits confirmation by further extended studies. Overall, the HLA-E-UL40 axis has an important impact on the level of HCMV replication in LTRs and may also provide a way for HCMV immune evasion.

## MATERIALS AND METHODS

### Patients.

In this study, 137 R^+^ or D^+^/R^−^ LTRs, transplanted at the Medical University of Vienna between 2013 and 2016, were included. From all patients, plasma samples were included (Table 1), and from 71 of these patients, formalin-fixed paraffin-embedded (FFPE) samples of the donor lung were available. All patients received induction therapy with 30 mg alemtuzumab (Berlex), and maintenance therapy with tacrolimus, corticosteroids, and mycophenolate-mofetil. All patients received antiviral (Val-)ganciclovir prophylaxis, R^+^ patients for 3 months and D^+^/R^−^ patients for 12 months, respectively. Patients were followed up by HCMV-qPCR, weekly for 2 months, monthly for 1 year after transplantation, and at longer intervals thereafter. Preemptive (Val-)ganciclovir treatment was initiated at HCMV-DNA levels of >1,000 copies/ml plasma. LTRs were followed up for 1 (R^+^) or 2 (D^+^/R^−^) years posttransplantation.

### Detection of HCMV-DNA and serology.

Viral DNA was isolated from plasma using NucliSens EasyMag (bioMérieux), eluted in 50 μl nuclease-free H_2_O, and measured by Cobas CMV monitor test (Roche, Branchburg, NJ, USA). HCMV-specific IgG antibodies were measured in plasma by enzyme-linked immunosorbent assay (ELISA) (Euroimmun).

### Genotyping.

DNA was isolated from plasma using NucliSens EasyMag and from FFPE donor lung samples using the FFPE DNA extraction kit (Favorgen). HLA-E*0101/0103 and HCMV-UL40 variants were determined as described before (11, 23).

### Next-generation sequencing.

In part, UL40 genotypes were analyzed by NGS. Nested primers were modified for MiSeq sequencing (Illumina). Amplicons derived from nested PCR were cleaned with the ExoSAP-IT kit (Thermo-Fisher, Waltham, MA, USA). For library preparation, 0.5 ng of products was used for index PCR, using Illumina indices with the HotStarTaq kit (Qiagen). NGS was performed according to the Nextera XP protocol (Illumina). The products were pooled equimolarly and sequenced for 150-bp paired ends. The paired-end reads were merged using CLC-Bio software to obtain single sequences for the whole amplified region. The sequences were then aligned and translated into amino acid sequences. For every sequence, the UL40 sequence of 9 amino acids was obtained and then quantified according to their sequence content.

### Cells and cell lines.

Peripheral blood mononuclear cells (PBMCs) were isolated by density gradient centrifugation (Ficoll Paque Plus; GE Healthcare) and screened for the presence of adaptive NKG2C^+^ NK cells as previously described (24). Adaptive NK cells were identified as CD56^+^ NKG2C^+^CD2^+^ CD57^+^ILT2^+^ Siglec-7^–^ NKp30^–^ NKG2A^–^ NK cells. CD56^+^ cells from donors with adaptive NK cell expansions were enriched by magnetically activated cell sorting (MACS) (CD56 MicroBeads; Miltenyi Biotec) and cryopreserved in fetal calf serum (FCS) (BioWest) containing 10% dimethyl sulfoxide (DMSO) (Sigma). K562–HLA-E cells (25) (provided by E. Weiss, Ludwig Maximilian University) and RMA-S–HLA-E cells (26) (provided by J. Coligan, NIH) were maintained in complete medium (RPMI 1640 containing glutamine and supplemented with 10% [vol/vol] FCS, 20 μM β-mercaptoethanol, and 100 U/ml penicillin-streptomycin; all Thermo-Fisher) in the presence of 400 μg/ml hygromycin B and 1 mg/ml G418 (both InvivoGen), respectively.

### HLA-E surface stabilization.

HLA-E surface stabilization was induced as described previously (13). In brief, target cells were incubated at a density of 2 × 10^6^ cells/ml for 16 h at 37°C with 0.1 to 300 μM peptides (Peptides&Elephants, Hennigsdorf, Germany) in 1 ml Opti-MEM (Thermo-Fisher). Peptide-pulsed cells were either stained for flow cytometric analysis of HLA-E surface expression (RMA-S–HLA-E) or washed with complete medium and used for *in vitro* stimulation (K562–HLA-E).

### *In vitro* stimulation of NK cells.

CD56^+^ MACS-enriched cells were quickly thawed at 37°C, washed, and rested in complete medium at 37°C. After 6 h, cells were stained with viability dye, anti-CD3, and anti-CD56; sorted for viable CD3^–^ CD56^dim^ NK cells on a FACSAria II (BD Biosciences); and rested overnight in complete medium. Purified NK cells were cocultured with irradiated (30 Gy) target cells pulsed with peptide (100 μM), in the presence of 100 μM synthetic peptides for 4 h, at an NK cell/target cell ratio of 2:1. Anti-CD107a was added at the start of the assay, and GolgiStop and GolgiPlug (both BD Biosciences) were added after 1 h. Cells were then washed with cold phosphate-buffered saline (PBS) followed by staining for flow cytometry.

### Flow cytometry.

Cell suspensions were stained with fluorochrome-conjugated antibodies (see Table S1 in the supplemental material), following established guidelines (24). For intracellular staining, cells were fixed and permeabilized using the Inside Stain kit (Miltenyi) according to the manufacturer’s instructions for cells in suspension. Dead cells were identified using the Zombie Aqua Fixable Viability kit (BioLegend) or Fixable Viability Dye eFluor780 (Thermo-Fisher). Data were acquired on an LSR Fortessa or FACSymphony (both BD Biosciences). FlowJo v10.6.1 was used for analysis.

10.1128/mBio.02996-20.4 TABLE S1 Conjugated antibodies used for flow cytometry. Download Table S1, DOCX file, 0.01 MB. Copyright © 2021 Vietzen et al. 2021 Vietzen et al. https://creativecommons.org/licenses/by/4.0/ This content is distributed under the terms of the Creative Commons Attribution 4.0 International license.

### Statistical analysis.

Distributions of the patient’s gender, genetic variants, and donor and recipient serostatus were compared by the χ^2^ test. Age was compared with the Kruskal-Wallis-test. Cytotoxicity assays were evaluated with paired *t* test or analysis of variance (ANOVA). Freedom from HCMV viremia was assessed with the Mantel-Cox test. *P* < 0.05 was considered significant. Statistical analyses were performed using IBM SPSS Statistics 24. The study was approved by the local ethics committees (EK-No.1687/2018, EA4/059/17).

### Data availability.

Newly generated sequence data were uploaded to GenBank (accession numbers: MW619533 to MW619623).
