# Supplementary figures and images for: Extent of Cytomegalovirus Replication in the Human Host Depends on Variations of the HLA-E/UL40 Axis
Source: mBio. 2021 Mar 16;12(2):e02996-20. doi: 10.1128/mBio.02996-20 (PMC8092275; doi:10.1128/mBio.02996-20)

# Figure S.1

## A

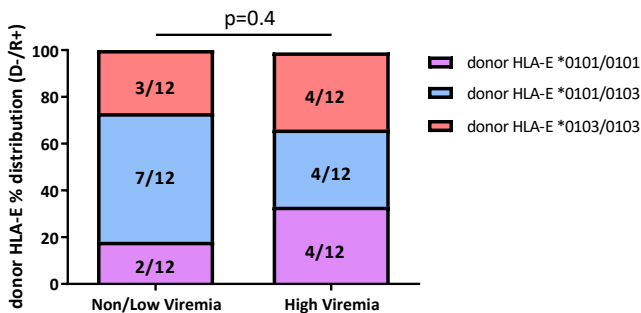

## B

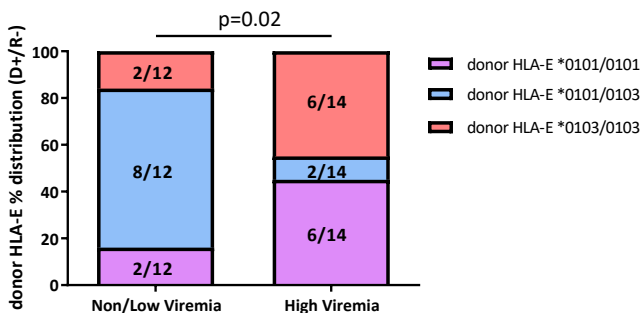

## C

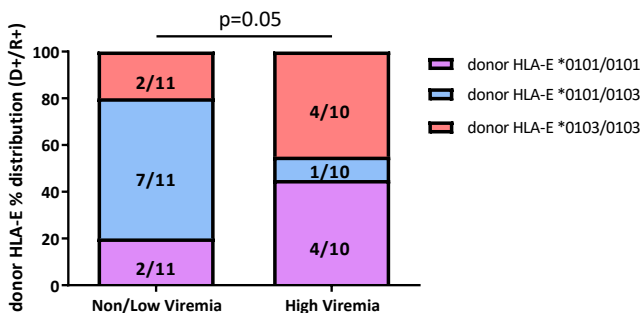

Supplement: FIG S1 [file mBio.02996-20-sf001.pdf]

**Figure S2**

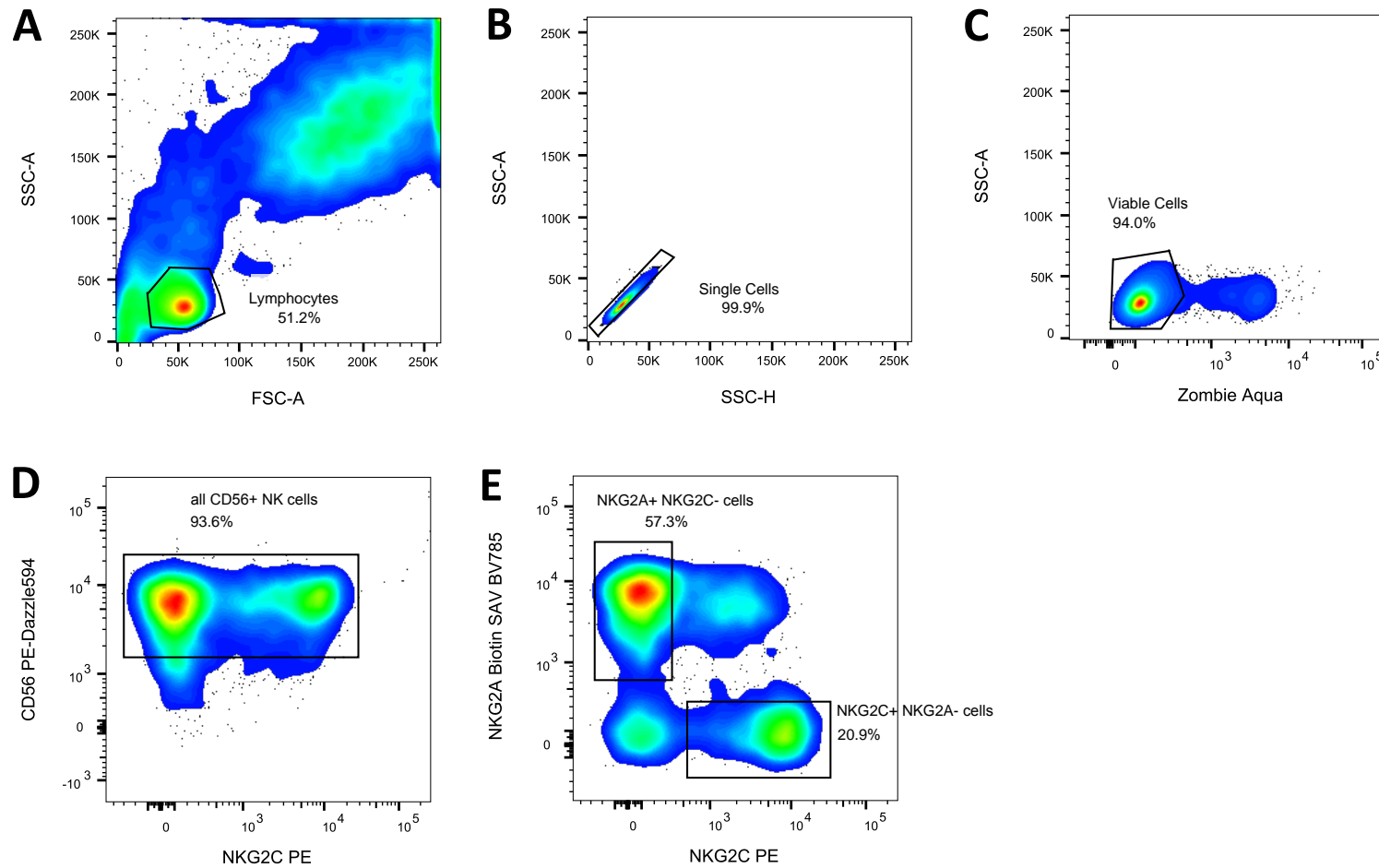

Supplement: FIG S2 [file mBio.02996-20-sf002.pdf]

Figure S.3

A

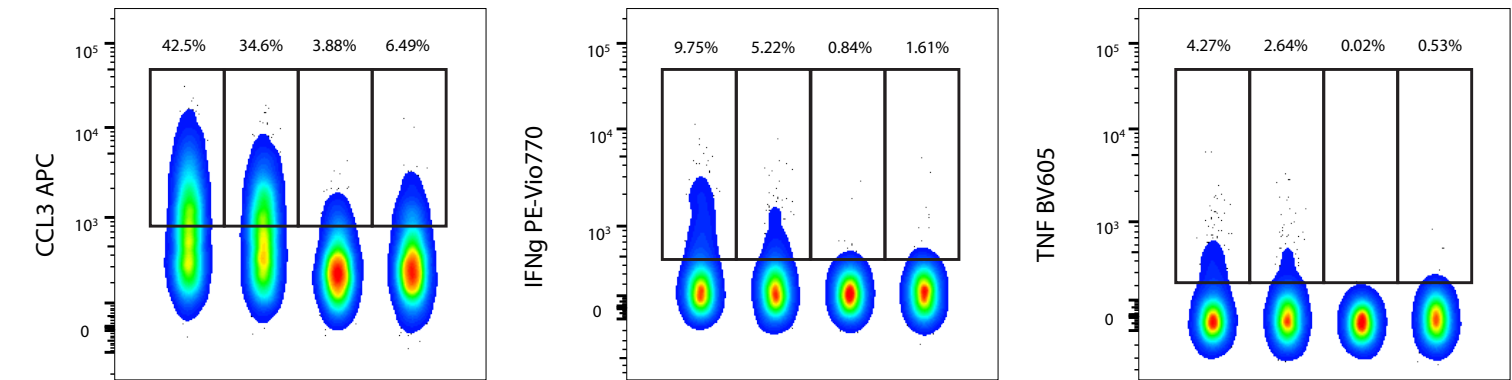

B

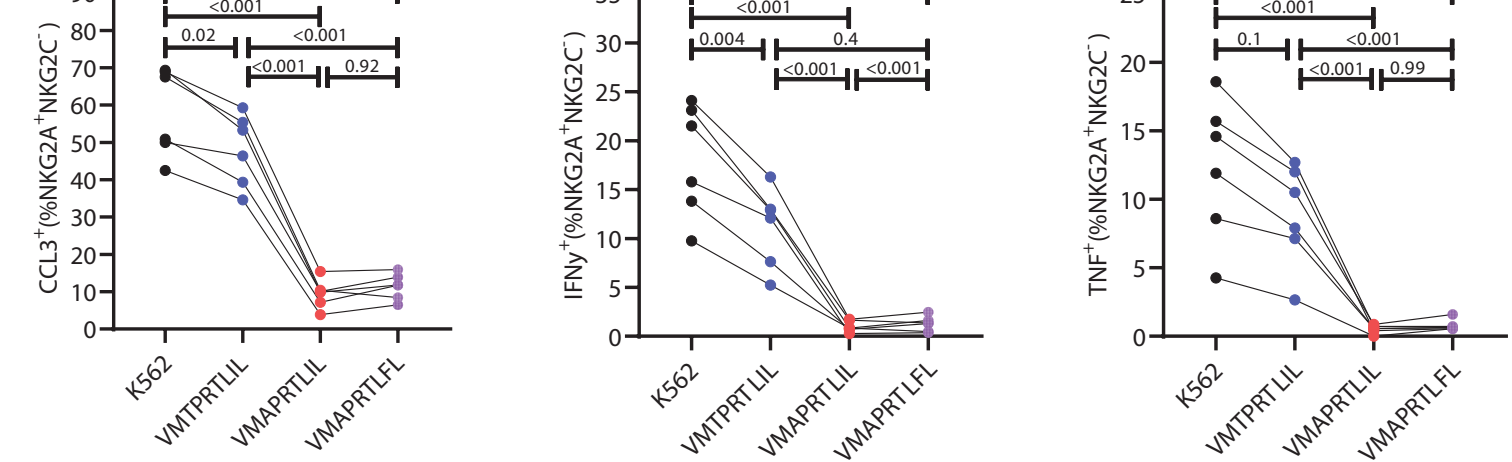

C

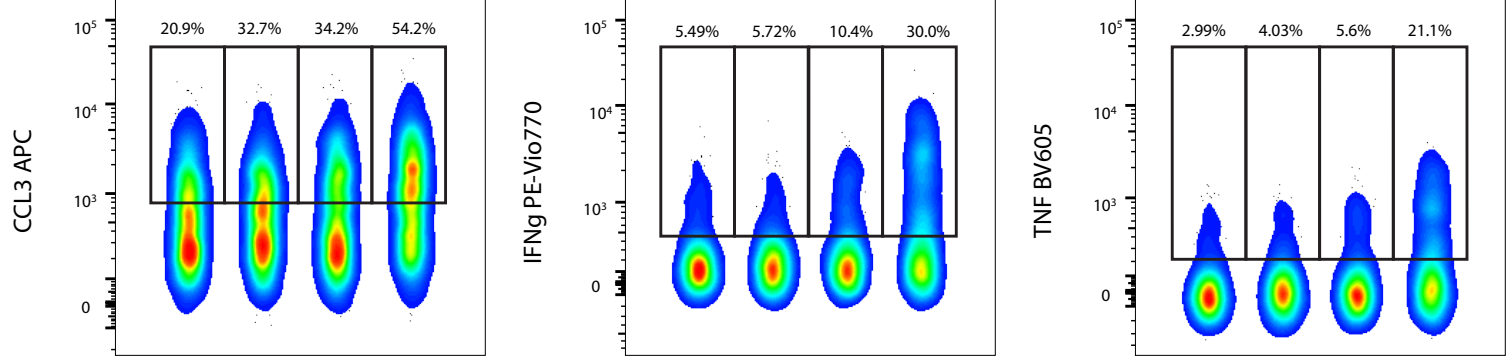

D

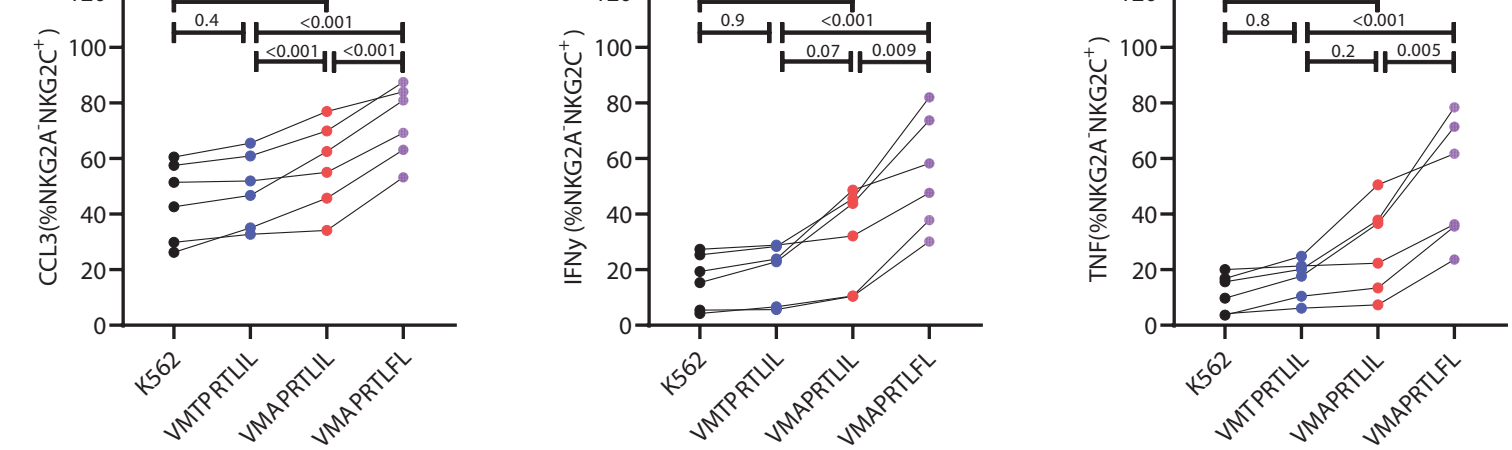

Supplement: FIG S3 [file mBio.02996-20-sf003.pdf]
