# Supplementary material for: Extent of Cytomegalovirus Replication in the Human Host Depends on Variations of the HLA-E/UL40 Axis
Source: mBio. 2021 Mar 16;12(2):e02996-20. doi: 10.1128/mBio.02996-20 (PMC8092275; doi:10.1128/mBio.02996-20)
Supplement: TABLE S2 [file mBio.02996-20-st002.docx]

Table S2: Predicted UL40- HLA-E Affinities

| Allele | Peptide | Smm_IC50 |
| --- | --- | --- |
| HLA-E*01:01 | VMAPRTLVL | 45 |
| HLA-E*01:01 | VMAPRTLLL | 45.10 |
| HLA-E*01:01 | VMAPRTLIL | 47.78 |
| HLA-E*01:01 | VMAPRTLFL | 51.79 |
| HLA-E*01:01 | VMTPRTLVL | 57.31 |
| HLA-E*01:01 | VMTPRTLLL | 57.44 |
| HLA-E*01:01 | VMTPRTLIL | 60.84 |
| HLA-E*01:01 | VMAPWTLVL | 62.12 |
| HLA-E*01:01 | VMAPWTLIL | 65.95 |
| HLA-E*01:01 | VMGPRTLIL | 98.91 |
| HLA-E*01:01 | VLAPRTLLL | 111.49 |
| HLA-E*01:01 | VMAPRILVL | 115.94 |
| HLA-E*01:01 | VMAPRILIL | 123.09 |
| HLA-E*01:01 | VIAPRTLIL | 133.73 |
| HLA-E*01:01 | VVAPRTLVL | 157.12 |
| HLA-E*01:01 | VMAPRSLLL | 178.74 |
| HLA-E*01:01 | VMAPRSLIL | 189.33 |
| HLA-E*01:01 | GMAPRTLLL | 259.55 |
| HLA-E*01:03 | VMTPRTLIL | 8674.41 |
| HLA-E*01:03 | VMTPRTLLL | 8674.41 |
| HLA-E*01:03 | VMTPRTLVL | 8674.41 |
| HLA-E*01:03 | VMAPWTLIL | 9252.09 |
| HLA-E*01:03 | VMAPWTLVL | 9252.09 |
| HLA-E*01:03 | VMAPRILIL | 9294.80 |
| HLA-E*01:03 | VMAPRILVL | 9294.80 |
| HLA-E*01:03 | VMAPRSLIL | 9359.23 |
| HLA-E*01:03 | VMAPRSLLL | 9359.23 |
| HLA-E*01:03 | VLAPRTLLL | 10238.59 |
| HLA-E*01:03 | VMAPRTLFL | 10333.32 |
| HLA-E*01:03 | VMAPRTLIL | 10333.32 |
| HLA-E*01:03 | VMAPRTLLL | 10333.32 |
| HLA-E*01:03 | VMAPRTLVL | 10333.32 |
| HLA-E*01:03 | VMGPRTLIL | 11021.48 |
| HLA-E*01:03 | VIAPRTLIL | 14003.61 |
| HLA-E*01:03 | VVAPRTLVL | 14003.61 |
| HLA-E*01:03 | GMAPRTLLL | 27116.90 |
