# Supplementary material for: Extent of Cytomegalovirus Replication in the Human Host Depends on Variations of the HLA-E/UL40 Axis
Source: mBio. 2021 Mar 16;12(2):e02996-20. doi: 10.1128/mBio.02996-20 (PMC8092275; doi:10.1128/mBio.02996-20)
Supplement: TABLE S1 [file mBio.02996-20-st001.docx]

Table S1: Conjugated Antibodies used for flow-cytometry:

| Antigen | Clone | Conjugate | Catalog No. | Manufacturer |
| --- | --- | --- | --- | --- |
| CD56 | HCD56 | PE-Dazzle594 | 318348 | Biolegend |
| CD3 | UCHT1 | Cy5 |  | In house |
| CD57 | HCD57 | Pacific Blue | 322316 | Biolegend |
| NKG2A | REA110 | Biotin | 130-113-564 | Miltenyi |
| NKG2C | RE205 | PE | 130-103-635 | Miltenyi |
| IFNγ | 45-15 | PE-Vio770 | 130-096-752 | Miltenyi |
| TNF-α | MAb11 | BV605 | 502936 | Biolegend |
| CCL3 | REA257 | APC | 130-103-630 | Miltenyi |
| CD107a | H4A3 | A488 | 328610 | Biolegend |
| Streptavidin |  | BV785 | 405249 | Biolegend |
